# Clinical Cases

**Published:** 1882-01

**Authors:** E. W. Berridge

**Affiliations:** London


### CLINICAL CASES.

E. W. Berridge, M. D., London.

*Rhus in Erysipelas.*—Jan. 14th, 1873, Captain ——, six and a half years ago kissed a child suffering from scarlatina, and felt it burn his lips. In a few weeks had boils on back; after this he became costive; once he fainted, fell and bruised his forehead, during the faint had an involuntary stool; soon erysipelas appeared on the bruised part of the forehead, and spread thence all over the face; it also appeared on the scrotum and adjoining surface of the penis, which suppurated. He has had four or five bad attacks of erysipelas since, and three or four slight ones. Since these attacks have come on, his sight for near objects has become impaired; formerly it was excellent, both for near and distant objects. The present attack came on January 12th, possibly excited by being in a new, damp house. About noon, he felt a shoot in left external orbital integuments; red, erysipelatous swelling began there and extended all over face, lids, forehead, chin and neck; also on scrotum. He never had it on neck and chin before. Vesicles form and exude a fluid which leaves yellow stains on the handkerchief. The affected parts burn and itch; *when lying down*, at each pulsation of the heart there is throbbing *centrifugally* in the inflamed integuments of the face and forehead. Any moisture to the skin brings out the erysipelas there, as it always has in these attacks. He must scratch the parts, which causes an "agonizing pleasure." The scratching of the scrotum causes sexual pleasure, and an escape of semen which wakens him. Photophobia, very restless all last night, no sleep, walking about, stamping, shaking arms, and striking about. To-day (January 14th), swelling increased; large yellow crusts from the discharge on chin; scrotum worse; lids closed from the swelling; the throbbing continues; the itching and burning are worse; the hands and feet are cold; pulse 50, intermitting in volume and rhythm; the burning and itching are worse from warmth. He has had the best allopathic treatment in former attacks, both in England and in India, but the physicians always made him worse by applying moisture and keeping him on low diet. All his allopathic physicians said it was an extraordinary case; one said he had examined all his books in reference to the case, but could do nothing for him. He informed me that once, when in London, he consulted Dr. Joseph Kidd, * the *professed* homœopath, for chronic dysentery. Dr. K. told him that he could cure him in ten days. Captain —— remained under his care for ten weeks without benefit, and afterwards cured himself by eating the pods of the Egyptian bean. He had consequently lost faith in *all* doctors, but his wife sent for me (in "an agony of despair," as Dr. Kidd would say).

This case, however puzzling to an allopath, was not so to a follower of Hahnemann. *Rhus* was unmistakably indicated, and I gave him *Rhus tox.* 2,000 (Jenichen) in water, a spoonful every hour in water until better; the first dose at 2.30 P. M. To take nourishing diet and wine and water (I should not *now* give wine, unless there was a great craving for it). At 8 P. M., I saw him again; he had had four doses; *improved after first dose;* itching, burning, and discharge much less; throbbing almost gone; pulse 60, more regular; less swelling; extremities still cold. *To stop medicine.*

---

*I am aware that it is contrary to etiquette to publicly mention another physician's name in connection with his blunders; but as Dr. Kidd did the same in his *Laws of Therapeutics*, he has no right to complain if *his* shortcomings are exposed. "With what measure ye mete, it shall be measured to you again."

Jan. 15th, 3 P. M. Slept well last night; much less swelling; only slight itching and burning; scrotum better; can bear the light; extremities still cold; pulse 72, regular; no other symptoms. *He says he has been relieved in one-third of the time usually occupied by such an attack.*

Jan. 22d. Steadily improved, and yesterday went out of doors for the first time, which he enjoyed, but felt very weak. It was a cold day. He felt better on coming into a very warm room. Afterwards went to bed in a very cold room. Soon the face felt hot; then followed itching and burning, and soreness at outer corner of left orbital integuments, which extended all over face, just as before, but less severely. Pulse 72, feeble. Scarcely any sleep last night, but could lie in bed. Scrotum as before, but not so bad. To test the accuracy of Carroll Dunham's statement that the action of *Rhus tox.* and *radicans* were the same, I gave *Rhus rad.* 200 (Leipzig) in water every two hours until better. (This I *now* see not to have been a true test, as the potencies and the periods of repetition differed in the two trials. *Rhus tox.* and *rad.* are now proved to be identical; and it will be seen that the 2000 acted better than the 200).

Jan. 23d. After 4 doses, decided relief, and stopped the medicine for a time, but took another dose at noon. Had itching on face *and head* last night, which disturbed sleep. To-day not much burning but some itching. Semen escaped as before. Can bear light. Pulse 72, rather feeble. Itching and burning are worse after food. Itching all over body more or less. In three or four days recovered completely, and had remained so when I saw him on Jan. 16th, 1874. He now has perfect confidence in HAHNEMANNIAN Homœopathy. After this I did not see him, as he went to Suez. I have had several letters from him, the last dated July 28th, 1879. He reports that since the summer of 1876 he has had several slight attacks of erysipelas, but *always* subdued them quickly with *Rhus.* On four or five occasions he applied Holman's liver pad for indigestion, and on each occasion it excited the erysipelas. He probably will require some well-indicated anti-psoric to *eradicate* the disease, but I have not had the opportunity of prescribing it for him. On May 31st, 1879, this gentleman, who had been driven away from homœopathy by Dr. Kidd's want of success, wrote to me as follows: "I wish I could come and have a few weeks' instruction from you. *I believe I have converted nearly half of Suez to Homœopathy.* I have had any amount of patients, of course, free, gratis, for nothing. My best cases of cure were an Arab from paralysis of face, with partial loss of sight; the other, English, an old-standing spleen case; and each of them with only one medicine."

There ought to be an opening at Suez for a Hahnemannian physician, but no *other* need apply, the patients being too well instructed.

